# The Newer, the More Secure? Standards-Compliant Bluetooth Low Energy Man-in-the-Middle Attacks on Fitness Trackers

**DOI:** 10.3390/s25061815

**Published:** 2025-03-14

**Authors:** Hannah Greß, Björn Krüger, Elmar Tischhauser

**Affiliations:** 1 Department of Mathematics and Computer Science, Phillips-University of Marburg, 35032 Marburg, Germany; elmar.tischhauser@uni-marburg.de; 2 Department of Epileptology, Medical Faculty, University Hospital Bonn, 53127 Bonn, Germany; bkrueger@uni-bonn.de

**Keywords:** fitness tracker, security, Bluetooth Low Energy, BLE, Internet of Things, IoT

## Abstract

The trend in self-tracking devices has remained unabated for years. Even if they record a large quantity of sensitive data, most users are not concerned about their data being transmitted and stored in a secure way from the device via the companion app to the vendor’s server. However, the secure implementation of this chain from the manufacturer is not always given, as various publications have already shown. Therefore, we first provide an overview of attack vectors within the ecosystem of self-tracking devices. Second, we evaluate the data security of eight contemporary fitness trackers from leading vendors by applying four still partly standards-compliant Bluetooth Low-Energy Man-in-the-Middle (MitM) attacks. Our results show that the examined devices are partially vulnerable against the attacks. For most of the trackers, the manufacturers put different security measures in place. These include short and user-initiated visibility and connectivity or app-level authentication to limit the attack surface. Interestingly, newer models are more likely to be attackable, underlining the constant need for verifying the security of BLE devices, reporting found vulnerabilities, and also strengthening standards and improving security awareness among manufacturers and users. Therefore, we finish our work with recommendations and best practices for law- and regulation-makers, vendors, and users on how to strengthen the security of BLE devices.

## 1. Introduction

The fitness tracker industry has been growing for years. The sales of wearables per year grew from 28.8 million to 506.6 million devices sold between 2014 and 2023. In 2023, the market size of fitness trackers was USD 53.94 billion, with an estimated growth to USD 290.85 billion by 2032 [1,2].

The motivation for self-tracking devices or apps is often the improvement of personal health or fitness. The corresponding tracking data produced by these devices or apps can be—and sometimes indeed is—being used outside of its intended purpose. This can be, for example, in medical research, either in the public sector with projects like DELPHI [3], All of Us [4] or Health-X dataLOFT [5], or in the private sector, like Apple [6,7] or Polar [8]. Furthermore, assured persons can profit from sharing their activity data with their health insurance to obtain certain benefits [9,10,11]. Other application areas of wearables consist of supporting medical treatment or training, for example, posture tracking for lower back pain [12,13]. Moreover, the way for *medical wearables* was paved within the EU by means of the Medical Device Regulation [14] for a deeper monitoring and analysis of diseases [15].

What the trackers all have in common is the recording, storage, and later transmission of sensitive data. The sensitive data include, for example, vital data of the user or their activity data, which may also contain location data [16,17,18,19,20]. Therefore, these sensitive data need to be adequately secured along the way through the whole ecosystem, which can be quite challenging given their complexity. Data breaches [21,22] or attacks on publicly available data [23], revealing both sensitive data about a user and their activity, show that their protection is indeed quite challenging and likewise highlight the need to improve the protection for these kinds of user data.

Over the past years, numerous studies were conducted focusing on the security of fitness trackers (and sometimes their companion app) to see how well the users’ data are protected. This work follows a similar approach, but puts the main focus on the Bluetooth connection between a fitness tracker and a phone. Our work surpasses the former Bluetooth analysis by conducting publicly known and still partly standards-compliant Bluetooth Low-Energy (BLE) MitM attacks, which can be conducted in the pairing phase of two legitimate BLE devices. The conducted attacks are the *Key Negotiation of Bluetooth (KNOB) Attack* [24], the *Fixed-Coordinate Invalid Curve Attack* [25], the *Secure Connections Downgrade Attack* [26,27] (3.H.2.3.1, 2.3.5.3, 3.5.1–3.5.2), and the *BT-Niño Man-In-The-Middle Attack (Bluetooth—No Input, No Output—Man-In-The-Middle Attack)* [28]. The investigated trackers were the Vantage M2 (2021), the Ignite 3 (2022), and the Vantage V3 (2023) from Polar, Xiaomi’s Mi Smart Band 6 (2021), 7 (2022), and 8 (2023), and two children’s fitness trackers from Garmin: the vívofit jr. 3 (2020) and the Garmin Bounce (2022) [29,30,31,32,33,34,35,36]. We chose Polar and Garmin as brands because their products are very popular among runners and cyclists. The children’s trackers were of specific interest because they target minors and, therefore, their data need particular protection. Lastly, we selected the bands from Xiaomi because they are a cheap and popular alternative to other trackers.

To the best of our knowledge, this is the first work applying several MitM attacks, which are possible due to design flaws in the Bluetooth specification, to fitness trackers. Other works [37,38] either only implemented some of these attacks without applying them to devices or launched only a single attack that targeted various devices, such as Xiaomi’s Mi Band and Mi Band 2 [24]. In addition to this, we conducted our research on fitness trackers that came to the market in recent years and that are still for sale, to show that it remains important to investigate the security of such devices, even if this has already been performed in previous works, as the following section shows. Moreover, we show that newer trackers from the same brand or even the same model are not necessarily more secure than their predecessor, even when the implementation flaws were previously reported to the manufacturers.

**Responsible Disclosures:** We contacted the vendors on 11 April 2023 to inform them about our findings for the Vantage M2, the Mi Smart Band 6, and the vívofit jr. 3. We reminded them on 8 May 2023 to ask if there were any updates yet. Garmin responded to our emails by saying that our “security concern has been reviewed and is being or has been addressed”. Xiaomi classified our findings as an “invalid vulnerability”. We did not obtain any information from Polar. We did not contact the manufacturers again after testing more of their devices in August 2024.

## 2. Related Work

Numerous studies have been conducted to evaluate the security of fitness trackers and their ecosystem, revealing that not all possible security measures were met in place. The parts of the ecosystem examined differ from work to work, be it only the Bluetooth connection between the examined fitness tracker and a mobile phone [17,18,19,39,40,41,42], the Bluetooth connection and the appropriate app for the fitness tracker [20], the Bluetooth and the WiFi connection [43,44], or the ecosystem as a whole [16,45,46,47,48,49,50]. An overview of the parts that count as in “the ecosystem” is presented in Section 3.2.

Out of the research papers covering the fitness tracker ecosystem as a whole for security analysis, we would like to cover [48] more in depth because the authors examined the Apple Watch—which has not been carried out a lot in literature—in addition to the seven other fitness trackers that they used for testing. Bluetooth-related security testing comprised controlled visibility and connectivity, the use of a resolvable private address (RPA), adequate mutual authentication between the tracker and the appropriate app, as well as tamper protection in both directions, i.e., the manipulation of the data acquired by the tracker or the tracker function itself. Clausing and Schiefer stated, for five out of seven fitness trackers, a good overall security level and especially highlighted the strong level of security for the Apple Watch. The same result for the Apple Watch can also be found in [42,44], underlining that the Apple Watch can be considered as a secure device. Another work to highlight in this category is [16]; the authors provide a good analysis of the fitness tracker ecosystem for each of its components, potential vulnerabilities, or privacy breaches, and finally conduct a security assessment on two fitness trackers based on their analysis.

The only work covering the Bluetooth connection between a fitness tracker and a smartphone and the appropriate app is the work by Gouda et al. [20]. Using the Bluetooth Host Controller Interface (HCI) snoop log of their mobile phone and the log files of the appropriate app, the authors extracted the Generic ATTribute (GATT) handles and payloads, with the aim of sending maliciously fabricated packets to the trackers under test. This was only successful for one out of four fitness trackers.

The paper by Fúster et al. [44] is worth mentioning as a paper covering the security analysis of the Bluetooth connection between the fitness tracker and the mobile phone, as well as the WiFi connection between the appropriate app and the server where the data are stored. Together with the research by Bin Azhar et al. [41], where they only tested the security of the Bluetooth connection of their fitness trackers, at works are of specific interest because the authors had a closer look on the security of fitness trackers targeted at minors. They both tested the predecessors of our examined vívofit jr. 3 (vívofit jr. [41] and vívofit jr. 2 [44]) and revealed severe security flaws. Fúster et al. [44] discovered for their examined devices a lack of mutual authentication between the wearable and the app, the use of insecure pairing methods (LE Legacy Pairing instead of Secure Connections), missing encrypted Bluetooth traffic, transmission of the Long Term Key (LTK) in plaintext, and static device addresses, making the devices vulnerable to tracking. The vívofit jr. 2 uses LE Legacy Pairing as an insecure pairing method, transmits its LTK in plaintext, and has a static device address. Bin Azhar et al. [41] used the Bluetooth device discovery service *BlueHydra*, built on top of Linux’s *bluez* library, which can discover all Bluetooth devices in range together with their Bluetooth version, Bluetooth address, and the received signal strength indicator (RSSI) value. The authors wrote a python script that was able to access the *BlueHydra* logs, with the aim of tracking a specific device given its Bluetooth address. In addition to that, the RSSI value was taken to estimate the distance between two devices, making the tracking even more precise. Knowing that minors might wear Bluetooth-enabled fitness trackers underlines the need to secure these devices as much as possible. Moreover, Zhang’s and Liang’s work [40] works showed how easy it can be to sniff and replay unencrypted data from some fitness trackers, which shows that secure devices should not only be for minors, but for all users, irrespective of their age.

The analysis of the papers that we mention in this section reveals that security flaws can be found in different devices, independently of the popularity of the tracker’s brand or its prize. Not all authors listed their devices under test, but, from those for which we had the information, we counted 33 different brands. The brand with the most tested devices was Fitbit, followed by Garmin and Xiaomi.

From the selected works, we identified three security issues regarding Bluetooth security, which are also summarized in Table 1. First, the permanent broadcast of the device’s static Bluetooth address or other static identifier leads to a certain identification of the wearer, makes the device vulnerable to tracking, or enables an attacker to generate a movement profile of the tracker—and, thus, also the wearer. In addition to that, unauthorized connection or pairing attempts can be made, which might lead to the access of sensitive data [16,19,39,41,42,43,44,45,46,47,48,49]. Second, the lack of a (strong) mutual authentication between the tracker and the mobile phone or appropriate app harbors the risk for unauthorized access to sensitive data or the injection of falsified data [44,47,48,50]. Third, end-to-end encryption may be missing, meaning that the lack of encryption from the device to the manufacturer’s server may enable an attacker to intercept sensitive data or inject falsified information [16,17,18,19,20,40,44,48].

What has not yet been considered are attacks that can be conducted during the pairing phase of a BLE device (in our case, a fitness tracker) and a mobile phone. Our work builds on this, and we tested eight contemporary fitness trackers from three popular brands—Polar, Garmin, and Xiaomi—against four MitM attacks that are still partly standards-compliant and possible due to design flaws in the Bluetooth specification.

## 3. Bluetooth and the Fitness Tracker Ecosystem

The following section starts with a short introduction to Bluetooth and the fitness tracker ecosystem. Afterwards, we give a brief outline of end-to-end encryption in the BLE context, we describe the applied BLE attacks in detail, and we finish with our experimental setup.

### 3.1. Bluetooth

The Bluetooth protocol consists of two core configurations, Bluetooth BR/EDR and Bluetooth Low Energy (BLE) added in version v4.0. Both operate on the unlicensed 2.4 GHz Industrial Scientific Medical (ISM) band. Bluetooth BR/EDR has 79 Radio Frequency (RF) channels (0–78) with a 1 MHz spacing, covering a range between 2400 MHz and 2483.5 MHz. In BLE, 40 RF channels with a 2 MHz spacing from 2402 MHz to 2480 MHz are used. The 40 channels are divided in 3 so-called *primary advertisement channels* (channels 37, 38, and 39) and 37 *general purpose channels* (channels 0–36). To minimize interference and fading, Bluetooth BR/EDR and BLE make use of frequency hopping [27] (1.A.1.2, 1.C.6.1, 2.A.1-2, 6.B.1.4).

### 3.2. Ecosystem

**Figure 1:**
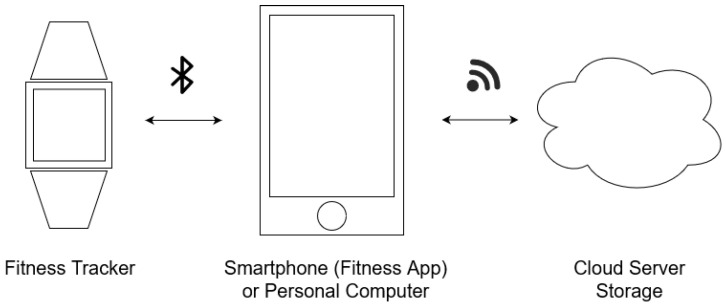
The fitness tracker ecosystem, based on [45,51] (p. 114).

As outlined in Figure 1, the fitness tracker ecosystem consists of the tracker itself on which its dedicated firmware is running, a smartphone or PC on which the vendor’s app or software for synchronization and displaying the data is installed, and the manufacturer’s server on which all the fitness data is stored. The fitness tracker records and stores the data on its internal memory until it is connected to the smartphone and app via BLE or to the PC via a USB cable for synchronization. The tracker uses the app or the software as proxy to transmit the recorded fitness data to the vendor’s server. In the ideal case, this information is end-to-end encrypted. To display the aggregated user data, the app requests these data from the server. Some manufacturers enable their user to also view the data in a web browser. For the communication between the app or software and the vendor’s server, a secure Hyper Text Transfer Protocol Secure/Transport Layer Security (HTTPS/TLS) connection is used [16,43,45,46] (p. 17), [49] (p. 3, p. 13, pp. 33–34).

### 3.3. End-to-End Encryption

End-to-End Encryption (E2EE) is an encryption between two endpoints—in our case, from the tracker to the vendor’s server. It prevents third parties from reading the data sent over this encrypted link [52].

### 3.4. Bluetooth Attacks and Vulnerabilities

#### 3.4.1. Key Negotiation of Bluetooth Attack

##### Attack Description

**Figure 2:**
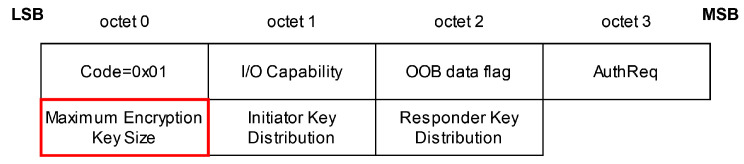
Key Negotiation of Bluetooth Attack: Pairing request or response package, based on [27] (3.H.3.5.1–3.5.2). The attacker changes the *Maximum Encryption Key Size* from 16 to 7 bytes. The modified octet is shown in red.

The *Key Negotiation of Bluetooth (KNOB) Attack* [24] is an MitM attack for Bluetooth BR/EDR and BLE during the first phase of the pairing procedure, the feature exchange phase. In the feature exchange phase, the two devices willing to pair exchange different values (see Figure 2) in the pairing request and pairing response to negotiate which pairing method (BR/EDR Legacy Pairing or Secure Simple Pairing (SSP) in Bluetooth BR/EDR and LE Legacy or Secure Connections (SC) in BLE) and which association model (Just Works, Numeric Comparison, Passkey Entry, or Out of Band (OOB)) they use to establish a secure connection. Since the message exchange is neither integrity protected nor encrypted at this point in the communication, an attacker can unnoticeably modify the transmitted packages. In case of the *KNOB* attack, the attacker alters the byte that specifies the length—more specifically, the entropy—of the long-term and short-term keys (LTKs and STKs, respectively) used for each session. The maximum key length is 16 bytes; in Bluetooth BR/EDR, the key length can be downgraded to 1 byte and, in BLE, to 7 bytes. These low entropy values enable an attacker to brute-force the LTK and STK of a Bluetooth connection, breaking the security guarantees on the link layer level provided by the Bluetooth standard. With the derived keys, an attacker can decrypt any ciphertext and modify or inject valid encrypted messages [24,27] (1.A.5, 2.H.1, 3.H.2.1, 3.H.2.3 and 2.3.4).

##### Countermeasures

Antonioli et al. [24] proposed two countermeasures: a legacy-compliant and a non-legacy-compliant one. The first proposal consists of requiring a higher minimum entropy value for the LTK, for example, 16 bytes, and every pairing should be aborted if this requirement is not fulfilled. The non-legacy-compliant proposal is to completely remove the key size negotiation from the feature exchange phase and to set the key size directly to a sufficiently high value, e.g., 16 bytes. An alternative could be to include an additional initial phase to establish and authenticate a key for protecting the integrity of the feature exchange phase and, therefore, the key size negotiation.

According to [53], for all devices using Bluetooth v5.0 and higher, a 16-byte key entropy is mandatory when using LE security mode 1, level 4. The LE security modes specify the security requirements of a device, a service, or a service request. LE security mode 1, level 4 is the highest possible security mode, enforcing pairing with authenticated LE Secure Connections with a 128-bit-strength encryption key [27] (3.C.10.2). BLE devices using using Bluetooth v5.0 or higher and LE security mode 1, level 4 by default may be vulnerable to the *KNOB* attack when falling back to LE Legacy pairing due to backwards compatibility.

#### 3.4.2. Fixed-Coordinate Invalid Curve Attack

##### Attack Description

**Figure 3:**
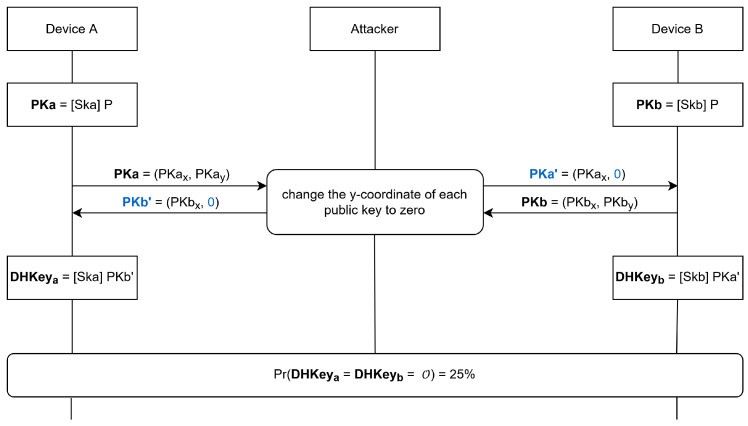
Fixed-Coordinate Invalid Curve Attack: Semi-Passive Attack, based on [25]. The attacker changes the y-coordinate of each public key to zero, resulting in a 25% chance that the attacker calculates the correct DHKey.

The *Fixed-Coordinate Invalid Curve Attack* [25] is an MitM attack conducted during the Elliptic-Curve Diffie–Hellmann (ECDH) key exchange during the paring of two Bluetooth devices, as shown in Figure 3. The attack makes use of the fact that the y-coordinate of the public key (PK) is not authenticated from the other device after transmission. Moreover, it was not mandatory to verify whether a given public key satisfies the curve equation or not before the attack became public. To exploit the missing authentication of the PK, the attacker sets the y-coordinate of both public keys to zero, transforming each public key into an elliptic-curve point with element order two, so that doubling the point yields the identity element (O). To calculate the shared ECDH key, denoted as Diffie–Hellman Key (DHKey) in Figure 3, the remote public key is multiplied with its own private key. The private key is a scalar, and since order-two points are equivalent to their own inverse, the multiplication of the private with the public key results either in the public key itself or in the identity element, depending on if the scalar is even or odd. This so-called semi-passive attack results in a 25% chance that the attacker can obtain the shared DHKey and result in a pairing success, as Table 2 demonstrates. In all other cases, the pairing is aborted. Compared to the fully active attack, the attacker needs only to be active and present during the generation of the shared DHKey.

**Figure 4:**
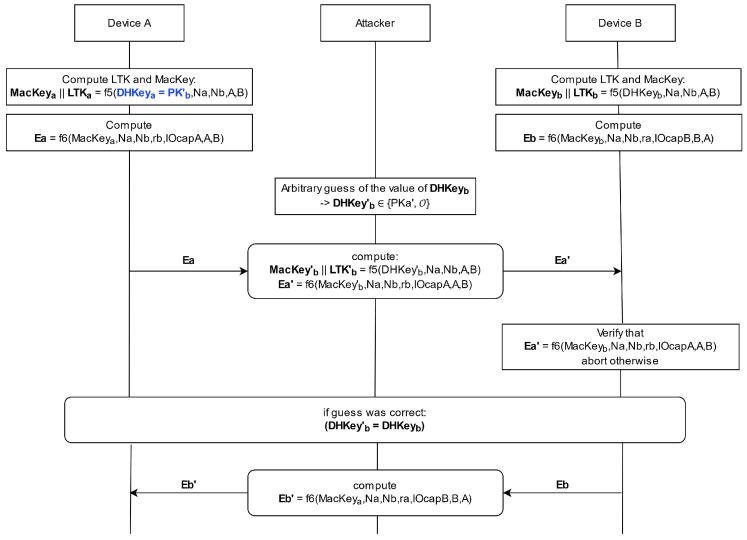
Fixed-Coordinate Invalid Curve Attack: The fully active MitM attack, based on [25]. The attacker calculates the value of $DHKey_{a}$ (here $PKb^{\prime }$) and guesses the value for $DHKey_{b}$, resulting in a 50% chance that the pairing succeeds.

**Figure 5:**
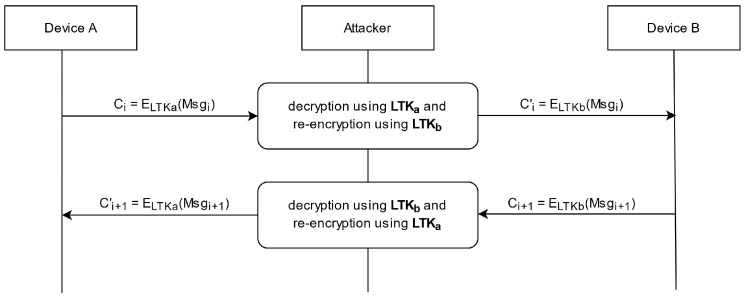
Fixed-Coordinate Invalid Curve Attack: The fully active MitM attack relay, based on [25]. The attacker needs to encrypt and decrypt every message before forwarding to the other device since the devices do not share the same long-term key.

The fully active attack extends the semi-passive one by not only intercepting during the ECDH key exchange, but also during the Message Authentication Code (MAC) and long-term key calculation, resulting in a 50% chance of a successful attack. As can be seen in Figure 4, the MAC and the long-term Key are calculated using function f5 with the shared DHKey and other public parameters as input. The attacker can compute the different results of function f5 using the four possible values for $DHKey_{a}$. In addition to that, they can calculate the value of Ea since the input parameters are either publicly known or can be retrieved with a small effort. The transmitted value of Ea reveals if $DHKey_{a}$ = $PKb^{\prime }$ or $DHKey_{a}$ = O. If $DHKey_{a}$ = O, the attacker can continue with the semi-passive attack. If $DHKey_{a}$ = $PKb^{\prime }$, as is highlighted in Figure 4, the attacker needs to guess the value of $DHKey_{b}$ to be either $PKa^{\prime }$ or O. The attacker calculates the value of $Ea^{\prime }$ and forwards it to the other device instead of the real value of Ea. If the guess was correct, the pairing can successfully be completed. If not, the pairing is aborted. The only drawback of the fully active attack is that the attacker needs to be online every time both devices want to communicate since they do not share the same long-term key. That means that the attacker needs to encrypt and decrypt every message before forwarding it to the respective device, as can be seen in Figure 5.

##### Countermeasures

To mitigate the *Fixed-Coordinate Invalid Curve Attack*, Biham and Neumann [25] proposed to test if a given ECDH public key satisfies the curve equation, meaning that the point is on the curve. Alternatively, if the base point *P* has a prime order *n*, it is also acceptable to test if the given public key satisfies [n]PK=O. This validation verifies that the public key does not have a small order.

The Bluetooth Special Interest Group (SIG) added these mitigations to their Bluetooth specification by indicating “[a] device shall validate that any public key received from any [Bluetooth Device Address (BD_ADDR)] is on the correct curve (P-192 or P-256)” [27] (2.H.7.1, 3.H.2.3.5.6.1) for Secure Simple Pairing (SSP) and Low-Energy Secure Connections (LE SCs), respectively. An erratum released in 2018 ensures that this mitigation is also applied to former versions (v2.1–v5.0), i.e., Bluetooth versions released before the attack was discovered [54].

#### 3.4.3. Secure Connection Downgrade Attack

##### Attack Description

**Figure 6:**
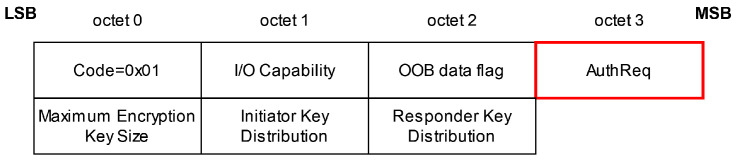
Secure Connection Downgrade Attack: Pairing request or response package, based on [27] (3.H.3.5.1–3.5.2). The attacker makes changes in the *AuthReq* octet. The modified octet is shown in red.

**Figure 7:**
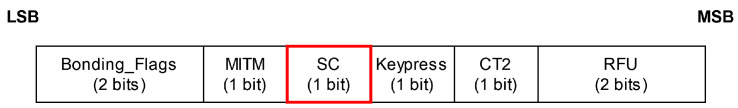
Secure Connection Downgrade Attack: Authentication requirements octet, based on [27] (3.H.3.5.1–3.5.2). The attacker sets the *SC* bit to zero, forcing *Legacy Pairing* instead of *Secure Connections*. The modified bit is shown in red.

With Bluetooth version 4.2, a new pairing mode was introduced, LE Secure Connections. Compared to the previous paring mode, known as LE Legacy Pairing, it uses the NIST P-256 elliptic curve for pairing, making the pairing procedure more secure. If two devices want to pair, they start with the feature-exchange phase, where they indicate i.a. if they support Secure Connections by setting the appropriate Secure Connections flag in the AuthReq byte as displayed in Figure 6 and Figure 7. If one or both of the devices have not set the flag, LE Legacy Pairing is performed.

As defined in the Bluetooth specification, the only secret value in LE Legacy pairing is the six-digit temporary key (TK), which may be displayed by one of the devices if the association model is Passkey Entry. If the association model is Just Works, the passkey is set to 000000. With this knowledge and having intercepted the other values exchanged during the pairing procedure, the attacker can calculate the STK and LTK if Just Works was used. In the case where the association model is Passkey Entry, the attacker can brute-force the TK because there are only $10^{6}=1,000,000$ possible values for it. The value for the TK can be determined within seconds using, for example, the open-source tool *crackle*: https://github.com/mikeryan/crackle (accessed on 28 January 2025).

Since the attacker is only active during the pairing procedure, the attack remains undetected by the victims because they can still communicate without the attacker being online during the whole communication and also during future connections [26,27] (1.D.1.1, 3.H.2.3.1, 2.3.5.3, 3.5.1–3.5.2).

##### Countermeasures

To prevent this attack, a device can enter the Secure Connections Only Mode, also called “FIPS Mode”. This means that only Federal Information Processing Standards (FIPS)-approved algorithms (AES-CMAC and P-256 elliptic curves) are used on the LE physical transport and “[a]uthenticated LE Secure Connections pairing with encryption using a 128-bit strength encryption key” [27] (3.C.10.2.1) (LE security mode 1, level 4). This implies that only Numeric Comparison, Passkey Entry, and Out of Band (OOB) can be used as the association model. Using the Secure Connections Only Mode can also have drawbacks since backwards compatibility with devices only supporting LE Legacy Pairing is not possible [27] (1.A.5.1, 5.3, 3.C.10.2.4, 3.H.2.3.1).

#### 3.4.4. BT-Niño Man-in-the-Middle Attack

##### Attack Description

**Figure 8:**
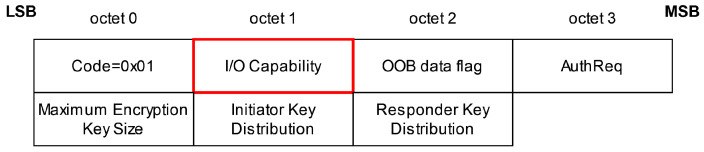
BT-Niño Man-In-The-Middle Attack: Pairing request or response package, based on [27] (3.H.3.5.1–3.5.2). The attacker changes the *I/O Capability* to *NoInputNoOutput*, forcing *Just Works* as association model. The modified octet is shown in red.

The *BT-Niño Man-In-The-Middle Attack (Bluetooth—No Input, No Output—Man-In-The-Middle Attack)* [28] is an attack first encountered for Bluetooth Secure Simple Pairing, but also works for BLE since the pairing processes are similar. As with the *KNOB* and *Secure Connection Downgrade Attack* described above, it is performed during the feature-exchange phase, but with the difference that the attacker modifies the I/O capability byte (see Figure 8), forcing the two devices to use the association model of their choice—in this case, Just Works. This association model does not provide any protection against MitM attacks.

Hyppönen and Haataja described three slightly different attack scenarios for their “Niño” attack. In all cases, the attacker owns two separate Bluetooth devices with modifiable Bluetooth addresses. Each attacker device clones the Bluetooth address and the user-friendly name (a 1–128-byte user-defined string to describe the Bluetooth device) of one of the victim devices in order to make the impersonation more plausible.

In the first scenario, the victim devices are already paired. The attacker jams the physical layer by either following the hopping sequence of the victims and sending randomly generated data in the time slots or by jamming the whole 2.4 GHz band. Consequently, the user may think that their Bluetooth devices do not work properly anymore and delete the stored link keys. After initiating a new pairing process, the attacker can manipulate the I/O capability information to downgrade the association model to Just Works. The rest of the pairing procedure continues as usual.

In the second and third scenarios, the victim devices are neither paired nor have they previously created a bond, so it is easier for the attacker to conduct the attack since the first phase, the disruption of the physical layer, can be skipped.

In the second scenario, the attacker observes the two victim devices and waits until one of them initiates a connection. In the third scenario, it is the attacker who starts by initiating the pairing with the victim devices at the same time. In both cases, the attacker modifies the I/O capabilities and remains passive for the remainder of the pairing process. When the attacker starts the connection, it may be possible to pair without needing the user’s confirmation, depending on the victim device’s implementation. When no user confirmation is needed, the attack can be performed completely silently.

Depending on the situation and the implementation of the victim devices, the attacker can perform one of three attack scenarios.

##### Countermeasures

The first countermeasure against the “Niño” attack can be carried out at the user interface level. Hyppönen and Haataja [28] proposed to force a device having a display to show the passkey, also called integrity checksum, even when the association model Just Works is used (Just Works is the default pairing method for some devices having I/O capabilities like a display). The user must compare this number to the number shown on the other device, in case it has a display. In their opinion, this forced comparison is a good compromise between usability and security. Another countermeasure is to use the private or silent security level. Using the private security level means that the device is non-discoverable and only accepts connections from future devices when their Bluetooth address is known. A device using the silent security level does not accept connections at all and just monitors the Bluetooth traffic. Other possible countermeasures consist of “increasing user awareness of security issues, minimization of transmit powers, careful selection of place where sensitive information is exchanged, and using additional security at the application level” [28]. Moreover, before accessing services or sensitive information, a re-authentication, independent from Bluetooth, should be performed.

To prevent MitM attacks in general, the authors propose to use an OOB channel with high, or at least good, usability.

### 3.5. Experimental Setup

We used the app *nRF Connect for Mobile* (https://www.nordicsemi.com/Products/Development-tools/nrf-connect-for-mobile, accessed on 28 January 2025) to sniff the Bluetooth traffic sent between the fitness tracker and the corresponding app to see whether the transmitted messages were end-to-end encrypted. In the recorded data, we searched for human-readable values or patterns to verify if E2EE was implemented.

To conduct the BLE attacks described in the section above, we used a single-board microcomputer Raspberry Pi 4 Model B with either a modified kernel or altered Bluetooth configuration. We used “Raspberry Pi OS with desktop” as our operating system and the kernel versions 5.15.y and 6.6.y for our experiments. The respective modifications for each attack can be found on Github: https://github.com/HannahGress/BLE_Attacks (accessed on 28 January 2025). We used the network protocol analyzer Wireshark to observe the exchanged packages between each fitness tracker and the Raspberry Pi to verify if the attack was successful and with which error code the fitness tracker replied in the case of failure. With each attack, we simulated a Man-in-the-Middle attack where the Raspberry Pi served as the MitM as well as the second pairing device in the connection. Therefore, the Raspberry Pi as the attacker was able to read, modify, and forge messages. Since all of the attacks are carried out during pairing, the messages transmitted between the sending and the receiving device are neither encrypted nor authenticated and are, therefore, vulnerable to sniffing and modification by a third party.

## 4. Results

We used eight different and very current fitness trackers for this experiment: the Vantage M2 (2021), the Ignite 3 (2022), and the Vantage V3 (2023) from Polar, Xiaomi’s Mi Smart Band 6 (2021), 7 (2022), and 8 (2023), and two children’s fitness trackers from Garmin—the vívofit jr. 3 (2020) and the Garmin Bounce (2022) [29,30,32,33,34,35,36].

### 4.1. Our Findings

#### 4.1.1. Vantage M2, Ignite 3, and Vantage V3

The Bluetooth traffic between the fitness trackers and the mobile phone can be sniffed, but they are partly human-readable when converted to UTF-8 and partly vendor-specific encoded, as the recorded data show patterns. This vendor-specific encoding is proven by an official document of Polar found after conducting the experiments [55].

The fitness trackers themselves are not vulnerable to the *KNOB* and the *Fixed-Coordinate Invalid Curve Attack*, but they show an unexpected behavior when the *Fixed-Coordinate Invalid Curve Attack* is launched by shutting down and restarting without showing anything to the user on their display. The fitness trackers are vulnerable to the *Secure Connection Downgrade* and the *BT-Niño Man-In-The-Middle Attack*, whereas they behave not as expected when launching the *Secure Connection Downgrade Attack* by setting the Secure Connections flag to 0 (false). This should only be the case when Secure Connections is not supported which is not the case with these fitness trackers. Thus, they somehow adapt their supported pairing mode to the supported pairing mode of the attacker [27] (3.H.3.5.1–3.5.2).

We did not investigate the unexpected behavior of the fitness trackers further since the focus our work has been the verification whether we can attack the selected fitness trackers or not.

#### 4.1.2. Mi Smart Bands 6, 7, and 8

The Mi Smart Bands 6, 7, and 8 have app-level authentication implemented for transmitting their data to the app, making it impossible for us to sniff the Bluetooth traffic with the *nRF Connect for Mobile* app. We did not investigate the Bluetooth HCI snoop log to see if end-to-end encryption is applied. However, Casagrande et al. [56] found out that the complete Bluetooth traffic of the Mi Band 6 is neither encrypted nor integrity protected. This might also be the case for its successors since the lack of end-to-end encryption has already been reported by Fereidooni et al. [57] in 2017 and nothing has been changed since then. Fereidooni et al. investigated the internet traffic sent between the vendor’s app and server with data coming from the tracker and not the Bluetooth traffic sent between the fitness tracker and the app. However, the authors could still conclude that there was no end-to-end encryption implemented because they could successfully modify the data, which were then accepted by the vendor’s server without any warning or error.

The Mi Smart Band 6 is not vulnerable to the *KNOB Attack*, but the Mi Smart Band 7 and 8 are. All three fitness trackers are robust against the *Fixed-Coordinate Invalid Curve Attack*, but not to the *Secure Connection Downgrade Attack*. Since their association model is Just Works by default, the *BT-Niño Man-In-The-Middle Attack* was not launched.

#### 4.1.3. Vívofit jr. 3 and Bounce

The Bluetooth traffic between the vívofit jr. 3 or the Bounce and the phone is not human-readable, but shows patterns which led to the assumption that it was proprietarily encoded and not end-to-end encrypted. This was also stated in [57] for the Garmin trackers that they examined (Vívosmart HR, Vívofit, and Vívofit 2).

Both devices are vulnerable to the *KNOB*, and the *BT-Niño Man-In-The-Middle Attack* and the Bounce also to the *Secure Connection Downgrade Attack*. The Bounce is not vulnerable to the *Fixed-Coordinate Invalid Curve Attack*, but shows an unusual behavior when launching the attack. After calculating and displaying the passkey, the tracker cuts off the connection between the attacking device and itself, but the passkey remains on the display and the user is not informed in any way as to what has happened. The vívofit jr. 3 uses LE Legacy Pairing for pairing; therefore, the *Fixed-Coordinate Invalid Curve* and the *Secure Connection Downgrade Attack* are not applicable.

### 4.2. The Vendors’ Countermeasures

Even if all devices show certain vulnerabilities, their vendors implemented auxiliary security measures to limit the practical impact of these attacks on the fitness trackers, but not on all.

Polar’s Vantage M2 is not visible by default—compared to the Ignite 3 and Vantage V3—and all three fitness trackers can be put into flight mode to be completely invisible and not connectable. In addition to that, pairing and connection need to be user-initiated by pressing a specific button on the tracker. The time interval during which the tracker is visible is also very short, about 30 to 60 s, decreasing the attack surface to a minimum.

Garmin’s vívofit jr. 3 is not visible by default and, as with the Polar devices, pairing and connection is user-initiated and its visibility is then limited to a short period of time. In contrast to that, the Bounce is always visible, connectable, and open for pairing, except when in flight mode, which is a feature hidden in the settings.

Being not visible, connectable, and pairable or having the ability to be put into flight mode are very simple but effective measures to protect the fitness tracker against hidden, unauthorized access by a third party.

Xiaomi’s Mi Bands are always visible, but the transmitted messages cannot be read since they are additionally authenticated on the app level. App-level authentication means that only the corresponding app can read the transmitted messages when the tracker is connected to the correct user account. In general, this is a good protection mechanism, but, as Casagrande et al. [56] showed, Xiaomi’s app-level authentication can be easily broken, at least until Mi Band 6. Therefore, both the Mi Band and the corresponding app could be connected to a malicious pendant without the user taking notice and the unencrypted and non-integrity-protected messages could be extracted or manipulated.

Regarding the end-to-end encryption, our analysis showed that none of the vendors implemented it. Polar’s and Garmin’s recorded messages show too much regularity to be considered encrypted. Moreover, we could extract some human-readable values out of Polar’s data, but not highly sensitive ones, underlining this assumption. Xiaomi did not implement encryption at all for its Mi Band 6, and this might also be the case for its successors since the lack of encryption has already been reported by Fereidooni et al. [57] in 2017 and nothing has been changed since then.

Table 3 summarizes our findings. As one can see, none of the trackers stand out negatively or positively regarding their level of security; each of them has its strengths and weaknesses.

## 5. Discussion and Future Work

### 5.1. Bluetooth Attack Summary and Discussion

All BLE attacks are publicly known and partially still standards-compliant, which means that, even when the Bluetooth stack is implemented correctly by following the Bluetooth specification, the devices are still vulnerable due to design flaws in the specification itself. Only for the *Fixed-Coordinate Invalid Curve Attack* and partly for the *KNOB* attack are the proposed mitigations established in the Bluetooth specification, and at least Polar and Xiaomi have implemented them appropriately on most of their trackers. However, regarding the *Secure Connection Downgrade* and the *BT-Niño Man-In-The-Middle Attack*, there are no mitigations described in the Bluetooth specification, leading vendors to not implement direct countermeasures against them. This shows that, despite a marked improvement in security awareness since the earlier studies on fitness trackers, manufacturers still do not focus as much on security as they should, usually choosing to only implement the absolutely necessary security measures, but not more. It is furthermore apparent that very few make the effort to keep up with published security research, only implementing and deploying countermeasures when explicitly required by a revision of the Bluetooth standard [53,54].

### 5.2. Implications of the Attacks

Due to a lack of security, either by the Bluetooth specification itself or the implementation of the vendors, the fitness trackers are theoretically vulnerable and it could be possible to sniff sensitive data. Practically, this is rarely the case because all attacks focus on the feature-exchange phase of the pairing between the device and the mobile phone of the user. Since the pairing is usually performed only once, because afterwards the devices are bonded and therefore know each other when connecting, the attacks can no longer be carried out. The only exception is if the attacker forces the two devices to pair again by disrupting the physical layer, as described in [28]. But the disruption of the physical layer leads to the question of the practicability of the approach, the effort one needs to put in, and if the user does not grow suspicious about the re-pairing. As such, all in all, one can say that these attacks pose a low risk to the users and their data, so they do not need to fear that their data are leaked to the public, even if they are not end-to-end encrypted. Nevertheless, vendors are advised not to passively acknowledge and tolerate the low risk; instead, they should strive to enhance the security of their products. This proactive approach aligns with the achievable measures required in this context. In the EU, this is enforced by the Cybersecurity Act [58] (2019) and the Cyber Resilience Act [59] (2022). The U.S. plans to do so, as mentioned in their National Cybersecurity Strategy [60], and California has already passed new IoT Security Laws in 2018, making it the first state in the U.S. [61]. On 13 April 2023, the American Cybersecurity and Infrastructure Security Agency (CISA), together with 17 U.S. and international partners, published a report with guidelines and best practices to make products secure by design [62]. This leads to the hope and assumption that these devices become more secure in the near future and that data breaches become less frequent and also less severe.

### 5.3. Recommendations and Best Practices

From the vendor’s side, it is recommended to apply end-to-end encryption from the fitness tracker to the server using a strong encryption mechanism like AES-256, which also provides integrity protection. Another alternative is the recently standardized *Ascon-Based Lightweight Cryptography Standards for Constrained Devices: Authenticated Encryption, Hash, and Extendable Output Functions* (NIST SP 800-232) [63]. These are designed for resource-constrained devices while providing robust security, efficiency, and flexibility. To better secure the Bluetooth connection, a good OOB mechanism should be used for pairing instead of the standard Bluetooth options. However, this should be well-designed because there is always a trade-off between security and usability. Using two-factor authentication would be an option too, but, in this case, there is again the question of usability and enough computing power on the tracker’s side. Another improvement would be a strong and mutual authentication mechanism between the tracker and the app. Lastly, good app-level authentication can be added to prevent other apps from reading the traffic sent between the two devices.

Regarding the role of regulation and industry standards, the Bluetooth specification should be improved to ensure the highest security and protection possible so that standards-compliant attacks like those we have shown can be avoided. This implies that the industry works hand in hand with security experts to enhance the quality of the protocol. In this case, the trade-off between security and backward compatibility has to be carefully evaluated. Another possibility is to redesign the Bluetooth specification, giving the security of devices the highest priority, maintaining backward compatibility with the current specification for a limited amount of time, and forcing the vendors to slowly make the transition to the newly designed one.

The users themselves have very limited to no possibility to improve the security of their Bluetooth device. The Bluetooth configuration is fixed, so all the values exchanged during the feature exchange phase are unchangeable. Therefore, the user is fully dependent on the vendor; the only thing that they can do is to keep the firmware updated—which is sometimes performed automatically—and to be distrustful when something seems suspicious, e.g., when the user needs to pair their device several times in a row.

The aforementioned recommendations and best practices refer not only to fitness trackers and their ecosystems, but to IoT devices in general. Since the market is growing, being in the Smart Home, Industry 4.0, or Internet of Medical Things (IoMT) areas, it is necessary that the manufacturers apply more security measures on their devices and systems. These include that vendors bring security experts along during the design and life process of a device, but end users should also be sensitive to security and potential vulnerabilities.

### 5.4. Future Work

As part of this work, eight contemporary fitness trackers have been analyzed with respect to four of the most generally applicable and practically relevant BLE attacks. The real-world application of the attacks showed tangibly that the security of these devices is good, but can and must be further improved since they are still vulnerable to certain attacks. As part of future work, it would be interesting to broaden the study by applying these attacks to fitness trackers from other vendors and to expand the security analysis by checking for Bluetooth vulnerabilities like device tracking [64,65,66], GATT Service and Characteristic authentication analysis [67], or others, as described in [53] or [68]. Lastly, a promising and equally important different line of research would be to extend the security analysis to other parts of the ecosystem, in particular the communication between the companion apps and the vendors’ backend services. All this could serve as a basis for a vulnerability assessment framework that could be used by the manufacturers to test their BLE devices for vulnerabilities prior to product launch.

## Figures and Tables

**Table 1:** Summary of all possible vulnerabilities and their consequences found in the literature.

Bluetooth Vulnerability	Related Work	Consequences
Permanent broadcast of static Bluetooth/device address or other static identifier	[16,19,39,41,42,43,44,45,46,47,48,49]	- Tracking/generation of movement profile - Unauthorized connection or pairing attempts → potential access to sensitive data - Tracker and, thus, user identification
Lack of (strong) mutual authentication between tracker and mobile phone/appropriate app	[44,47,48,50]	- Unauthorized access to sensitive data - Injection of falsified data
Missing end-to-end encryption	[16,17,18,19,20,40,44,48]	- Sniffing of sensitive data - Injection of falsified data

**Table 2:** Fixed-Coordinate Invalid Curve Attack: The possible values for $DHKey_{a}$ and $DHKey_{b}$, based on [25].

${\mathrm{DHKey}}_{a}$	${\mathrm{DHKey}}_{b}$
*∞*	*∞*
*∞*	$PKa^{\prime }$
$PKb^{\prime }$	*∞*
$PKb^{\prime }$	$PKa^{\prime }$

**Table 3:** Summary of the Bluetooth attack results on selected fitness trackers. ✗ signifies that the device remained unaffected, while ✓ indicates vulnerability. ‘?’ means that this could not be tested with the methods used and ‘-’ indicates that this attack is not applicable to the tracker.

Brand	Device	E2EE	KNOB	FCIC	SCD	Niño
Polar	Vantage M2, Ignite 3, Vantage V3	✗	✗	✗	✓	✓
Xiaomi	Mi Smart Band 6	✗ ^a^	✗	✗	✓	- ^b^
	Mi Smart Band 7 and 8	?	✓	✗	✓	- ^b^
Garmin	vívofit jr. 3	✗	✓	- ^c^	- ^c^	✓
	Bounce	✗	✓	✗	✓	✓

^a^ Based on the findings of Casagrande et al. [56]. ^b^ The attack was not launched because the attack modifications could already be found in the tracker’s Bluetooth configuration. ^c^ The attack could not be launched because the tracker does not support the attackable pairing mode.

## Data Availability

The respective modifications for each attack can be found on Github: https://github.com/HannahGress/BLE_Attacks (accessed on 28 January 2025).

## Abbreviations

The following abbreviations are used in this manuscript:

E2EE	End-to-end-encryption
KNOB	Key Negotiation of Bluetooth Attack
FCIC	Fixed-Coordinate Invalid Curve Attack
SCD	Secure Connection Downgrade Attack
Niño	BT-Niño Man-In-The-Middle Attack
